# Prevalence and Predictors of Normal-Weight Obesity among Women

**DOI:** 10.3390/nu16162579

**Published:** 2024-08-06

**Authors:** Olga I. Parfenteva, Nikolay A. Kulemin, Elvira A. Bondareva, Ildus I. Ahmetov

**Affiliations:** 1 Department of Molecular Biology and Genetics, Lopukhin Federal Research and Clinical Center of Physical-Chemical Medicine of Federal Medical Biological Agency, 119435 Moscow, Russia; parfenteva.olga@mail.ru (O.I.P.); bondareva.e@gmail.com (E.A.B.); 2 Laboratory of Genetics of Aging and Longevity, Kazan State Medical University, 420012 Kazan, Russia; 3 Research Institute for Sport and Exercise Sciences, Liverpool John Moores University, Liverpool L3 5AF, UK

**Keywords:** normal weight obesity, subcutaneous fat thickness, A-mode ultrasound, eating habits

## Abstract

The present study aimed to (a) assess normal-weight obesity (NWO) and general obesity prevalence among women of different ages residing in urban areas, (b) evaluate subcutaneous fat thickness (SFT) in women with NWO, (c) establish SFT cutoff points for distinguishing NWO, and (d) explore eating habits linked to NWO. This cross-sectional study with 184 women aged 18–65 with NWO, normal weight without obesity (NWNO), overweight and general obesity included evaluation of body composition, SFT assessment using 2.5 MHz A-mode ultrasound (ISAK protocol, 7 sites) and lifestyle inquiries. The curvilinear relationship between body fat and BMI rendered BMI an unreliable indicator of adiposity in women with normal weight (BMI < 25 kg/m^2^). Almost 30% of women with a high body fat percentage (BFP ≥ 30%) were misclassified when BMI was used to measure adiposity. The overall obesity prevalence defined by BFP was almost four times higher than that defined by BMI (56.0 vs. 18.0%, *p* = 1 × 10^−4^). Women with NWO, overweight and general obesity shared a similar SFT profile and eating habits, setting them apart from those with NWNO. The mean SFT was the most reliable NWO predictor, with a threshold set at 12 mm equal to the 66th percentile. Mean SFT accurately classified 85% of women with NWO. While age did not significantly affect subcutaneous fat accumulation, total fat levels increased with age (R^2^ = 0.07 and R^2^ = 0.19, *p*_adj_ = 0.1 and *p*_adj_ = 9 × 10^−4^). Higher NWO prevalence in middle-aged women was linked to age-related increase in fat mass and decrease in fat-free mass. Engaging in regular physical activity and reducing snack consumption effectively countered age-related changes in body composition (*p*_adj_ < 0.05). Women under 45 years who consumed sweet bakery items, fast food, and snacks more frequently showed higher BFP and NWO status (*p*_adj_ < 0.05). Prevention strategies should focus on monitoring body composition and promoting healthy behaviors, particularly among young women transitioning into adulthood and women over 45 years.

## 1. Introduction

Normal-weight obesity (NWO) is characterized by elevated total body fat (TBF) levels, typically exceeding 30% in women and 25% in men, despite having a normal body mass index (BMI) [1,2]. Increased TBF, waist circumference (WC), and BMI (≥30 kg/m^2^) are indicators of general obesity. Both conditions are associated with an abnormal metabolic profile related to excessive total, visceral, and subcutaneous body fat, suggesting potential health risks and the necessity for effective prevention strategies [3]. Individuals with NWO exhibit reduced insulin sensitivity, increased visceral adiposity, a more atherogenic lipid profile, and elevated blood pressure [3]. The combination of these factors increases risks of hyperinsulinemia, insulin resistance, type II diabetes, hypertriglyceridemia, and cardiovascular disease [1,3,4,5,6,7,8,9,10]. Identifying subjects with NWO is crucial, as they may experience hidden health risks despite normal weight. Inclusive health assessments, including body composition analysis and lifestyle inquires, are essential for individuals not only with overweight and general obesity but also with normal weight (BMI < 25 kg/m^2^). A fast and convenient ultrasound method for evaluating TBF and subcutaneous body fat can effectively be utilized for this purpose. It shows high reliability and validity, allowing precise measurement of subcutaneous fat thickness (SFT) and offering valuable insights into body composition and fat distribution in individuals with NWO [11,12,13,14].

Identifying factors that influence NWO risk enables the creation of targeted interventions and effective public health strategies, addressing this increasing health concern more efficiently. Urban “obesogenic” environments contribute to chronic energy imbalance, promoting increased adiposity through factors like easy access to unhealthy foods and limited opportunities for physical activity, exacerbating obesity-related health issues [15]. Men and women may react differently to ‘obesogenic’ environmental cues, which can significantly affect their dietary habits, physical activity level, and NWO prevalence [15]. Despite women generally being more concerned about weight maintenance and diet, they face a higher risk of developing NWO and general obesity compared to men [2,16]. NWO prevalence in women is two to six times higher than in men [2]. This disparity prompts research to investigate dietary habits and physical activity in women with NWO. Numerous studies reported NWO prevalence and its association with obesity risk factors; however, the results were inconsistent [17,18,19,20,21].

The present study aimed to (a) assess NWO and general obesity prevalence among women of different ages residing in urban areas, (b) evaluate SFT in women with NWO, (c) establish SFT cutoff points for distinguishing NWO, and (d) explore eating habits linked to NWO.

This study proposes that women with NWO have a body composition and SFT profile similar to those with overweight and general obesity, possibly influenced by their eating habits.

## 2. Materials and Methods

### 2.1. Data Collection and Inclusion Criteria

**Figure 1:**
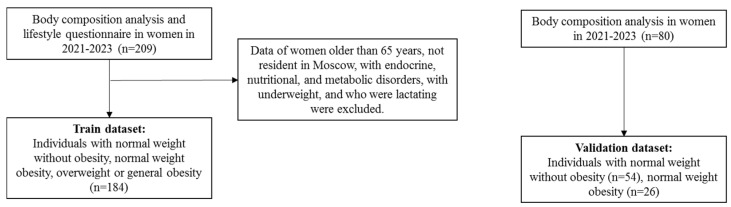
The inclusion and exclusion criteria used to form the training (N = 184) and validation (N = 80) datasets.

This cross-sectional study with women residing in urban agglomeration included one-time anthropometric measurements and lifestyle habit inquiries. Participants were recruited via social networks. Inclusion criteria comprised a specific age range (18–65 years), residency in Moscow, BMI more than 18.5 kg/m^2^, absence of endocrine, nutritional, and metabolic disorders, no lactation, and no missing data (Figure 1). The procedures took place between years 2021 and 2023 at the Lopukhin Federal Research and Clinical Center of Physical-Chemical Medicine of the Federal Medical Biological Agency of the Russian Federation. The final dataset included 184 women.

### 2.2. Nutritional Status Groups and Obesity Prevalence

Differentiation criteria included body fat percentage (BFP, %) and BMI. The maximum BFP in women with BMI < 25 kg/m^2^ aged 18–23, 24–29, 30–44 and 45–65 years were 31.1%, 33.0%, 36.1%, and 37.1%, respectively. The lowest recorded BFP for women with overweight was 29.3% in late adolescents, 31–32.0% in emerging and established adult women and 33.0% in middle-age women. The threshold for excess adiposity was set at 30% across all age categories. This threshold had been suggested in previous recommendations [1].

Women were classified into four groups: normal weight without obesity (NWNO: BFP < 30% and BMI < 25 kg/m^2^, N = 80), NWO (BFP > 30% and BMI < 25 kg/m^2^, N = 30), overweight (OW: 30 > BMI ≥ 25 kg/m^2^, N = 40), and general obesity (GO: BMI ≥ 30 kg/m^2^, N = 34). BMI and BFP ranged from 18.5 to 48 kg/m^2^ and from 15.6 to 41.5%.

### 2.3. Age Groups

The sample was divided into four age groups: late adolescent (18–23 years), emerging adulthood or pre-adults (24–29 years), established adulthood (30–44 years), and middle age (45–65). The training dataset contained 28 late adolescents, 34 pre-adults, 78 established adult women, and 44 middle-aged women. The validation dataset included 37 late adolescents, 6 pre-adults, 30 established adults, and 7 middle-aged women.

### 2.4. Body Composition Analysis

The ISAK protocol was implemented to assess body composition and anthropometric measurements [22]. Body weight was measured using a Seca 813 electronic floor adult scale with an accuracy of 0.1 kg (Seca, Hamburg, Germany). Measurement of body height was taken using laser KAFA device with accuracy of 0.1 cm (KAFA tools, Moscow, Russia). Body composition was assessed using 2.5 MHz amplitude modulation (A-mode) ultrasound scanner BodyMetrix™ (BodyMetrix Pro System BX2000; Livermore, CA, USA). Calibration was performed during the first use of the BodyMetrix System [23]. Measurements adhered to the manufacturer’s guidelines, utilizing 5 mm of gel and ensuring no pressure to prevent compression of subcutaneous fat, while maintaining good contact for accurate results. A-mode scans generate waveforms with peaks at tissue interfaces, where subcutaneous fat and muscle meet [23]. Subcutaneous fat thickness (SFT) was measured at seven sites according to ISAK protocol (chest, subscapular, mid-axilla, triceps, abdomen, suprailiac, and thigh), with each measurement repeated three to five times [22,23,24]. Total body fat mass (FM, kg), body fat percentage (BFP, %), and fat-free mass (FFM) were predicted based on SFT measurements using seven-site Jackson–Pollock formula [24]. All calculations were performed using BodyViewProFit Software (v. 1.0.0.90) [23]. Prior studies in Russia validated ultrasound measurements’ reliability, highlighting their effectiveness and accuracy in evaluating body composition [13]. Mean SFT was calculated based on all seven measurements.

### 2.5. Consumption of ‘Unhealthy’ Food

Women reported their frequency of consuming unhealthy foods. The model incorporated 11 predictor variables related to unhealthy eating behaviors. These were as follows: sticking to a healthy lifestyle and eating behavior (Yes/No: N = 94/90); reviewing food composition (Yes/No: N = 77/107); meal skipping (Yes/No: N = 20/164); meal frequencies (1–2, 3 and more); snacking (Yes/No: 132/52); consuming sweet beverages like soda, fruit juice, tea and coffee with sugar (Yes/No: N = 82/102); consuming mass-produced “fast-food” (Yes/No: N = 103/81),; consuming sweet bakery items (Yes/No: N = 129/55); consuming ready-to-eat sauces (Yes/No: N = 111/73); and using meal delivery services (Yes/No: N = 75/109). Participants were also asked about regularity of physical activity (Yes/No: N = 52/132). “Yes” indicates regular behavior no less than once a week. “No” means irregular behavior (several times per year) or avoidance. Participants completed the questionnaire prior to examination. Participants were asked about any dietary restrictions (vegan food, no sugar, no lactose, calorie restriction, etc.), health conditions, procedure limitations, number and age of biological children, lactation, involvement in professional sport, ethnicity of parents, education, occupation, duration of residency in Moscow, and recent weight change. The final dataset included women aged 18–65 years, residing in Moscow for at least five years or since birth, with stable weight, no professional sports involvement, and at least one Russian parent. Most women held higher education degrees. Dietary habits were assessed using the Nutrilogic program (v. 3.20), facilitating the collection of dietary data (https://nutrilogic.ru/; accessed on 15 June 2024).

### 2.6. Validation Dataset

The cutoff points were validated using an external dataset, ensuring the reliability and generalizability of the findings. The dataset included data on SFT at seven sites, BFP, FM, FFM, age, and nutritional status. The data were obtained during routine examination. All measurements were performed using the same equipment and personnel. Examination was performed in 2021–2023 at the Lopukhin Federal Research and Clinical Center of Physical-Chemical Medicine of the Federal Medical Biological Agency of the Russian Federation. Data integrity was maintained with no duplicated entries. The validation dataset contained 26 women with NWO aged 18–65 and 54 women with NWNO aged 18–65. The validation dataset was not used to train data.

### 2.7. Statistical Analysis

Data analysis was performed in R environment (version 4.0.3), easyROC program (http://biosoft.erciyes.edu.tr/app/easyROC/; accessed on 15 June 2024), and Past (version 4.16). Table 1 presents statistical procedures applied in the current study.

## 3. Results

### 3.1. Age-Related Differences in Prevalence of Exceeding BFP and BMI

**Figure 2:**
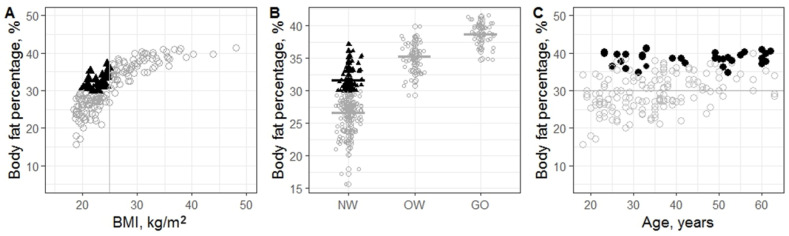
Distribution of BFP and BMI. (**A**). Relationship of BFP and BMI across the entire sample. Black triangles indicate NWO cases. (**B**). Distribution of BFP in women with normal weight (NW, BMI < 25 kg/m^2^), overweight and general obesity. Grey and black crossbars indicate median values. Black triangles indicate NWO cases. (**C**). Changes in BFP from younger to older groups. Black circles indicate cases of general obesity (BMI ≥ 30 kg/m^2^).

A strong curvilinear relationship (linear: R^2^ = 0.71 vs. curvilinear: R^2^ = 0.78, *p* = 1 × 10^−15^) was found between BFP and BMI (Figure 2A). The relationship between BFP and BMI was less pronounced at lower BMI. BFP varied from 15.6 to 37.1% in women with BMI less than 25 kg/m^2^. BFP variation (from 29.3 to 41.5%) was lower in women with overweight and general obesity. BMI did not accurately reflect adiposity in women with BMI < 25 kg/m^2^, as many women with high BFP were not classified as overweight or obese based on their BMI (*p* = 1 × 10^−4^). High BFP variability in women with BMI < 25 kg/m^2^ may indicate NWO cases.

More than a half (57%) [95% CI: 50–64%]) of women had BFP ≥ 30% (Figure 2). Among them, only 35% [95% CI: 20–41%] had a BMI greater than 25 kg/m^2^ (Figure 2A,B). Elevated body fat was observed even in women with normal BMI (Figure 2A, black triangles indicate NWO). One in four women (27% [95% CI: 18–34%]) with normal BMI had BFP exceeding 30%.

The overall NWO prevalence was 16% [95% CI: 6–35%]. Comparable rates of NWO and general obesity prevalence were observed among women under 45 years (Table 2). Middle-aged women stood out from other age groups in terms of general obesity prevalence. Lower NWNO prevalence in middle-aged women was attributed to higher NWO and general obesity prevalence. After adjusting for multiple testing, all differences were no longer significant, which may have been due to the small sample size.

In summary, NWO cases were observed in all age groups. The prevalence of NWO slightly increased from younger to middle-age groups. Increased prevalence of NWO in middle-age women might be associated with negative body composition changes associated with age, specifically a decrease in fat-free mass and increase in fat mass.

### 3.2. Body Composition Variation in Women across Age Groups

The study found notable variations in all measured parameters according to age (Table 3). The most significant differences in body fat accumulation were observed between middle-age women and younger subjects. Middle-aged women had higher BMI linked to higher BFP (rho = 0.86 [95% CI: 0.75-0.92], *p*_adj_ = 4 × 10^−11^). They showed 1.5-fold differences in BFP (*p*_adj_ = 2 × 10^−5^ _(18–23 vs. 45–65)_, 2 × 10^−5^ _(24–29 vs. 45–65)_, 4 × 10^−4^ _(30–44 vs. 45–65)_) and FMI (*p*_adj_ = 4 × 10^−3^ _(18–23 vs. 45–65)_, 4 × 10^−3^ _(24–29 vs. 45–65)_, 4 × 10^−3^ _(30–44 vs. 45–65)_) compared to other groups, despite having more or less similar FFMI (*p*_adj_ > 0.05) and FFM (*p*_adj_ > 0.05).

A steady linear increase in BFP (0.23% annually, *p*_adj_ = 2 × 10^−11^) was revealed from younger to older groups, suggesting shifts in body composition with age (Figure 2C). No significant association was found between FFMI, FFM and age. As age increased, the discrepancy in underestimating obesity based on BMI also increased, indicating a potential influence of age on the inaccuracies of BMI in estimating adiposity (Figure 2C, black circles indicate BMI ≥ 30 kg/m^2^).

SFT in chest (β = 0.12, *p*_adj_ = 4 × 10^−3^), subscapular (β = 0.07, *p*_adj_ = 8 × 10^−4^), and mid-axilla (β = 0.19 mm, *p*_adj_ = 3 × 10^−5^) regions increased from younger to older age groups (Table 2). A slight increase may indicate potential age-related changes in body fat distribution. No significant variations in SFT measurements for the triceps (*p*_adj_ = 0.46), abdomen (*p*_adj_ = 0.35), suprailiac (*p*_adj_ = 0.07), and thigh (*p*_adj_ = 0.14) regions among the participants of various ages were observed, suggesting consistency in these regions across the sample. After adjusting for nutritional status, the impact of age on SFT changes diminished, suggesting that diet and other factors play a crucial role in body fat accumulation.

### 3.3. Age-Related Changes in Lifestyle and Its Effect on Body Composition

Differences in lifestyle habits were noted across age groups, indicating varying behaviors and choices that may influence body composition and obesity prevalence (Table 4). Women under 45 years had unhealthier diets. They consumed sweet bakery items, sweet beverage and fast food and also skipped meal more frequently. Women over 45 years old were less physically active.

**Figure 3:**
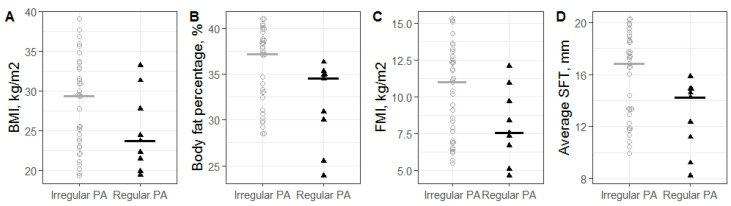
Distribution of BMI, BFP, FMI, average SFT in groups of middle-aged women with irregular and regular physical activity. (**A**). Changes in BMI. (**B**). Changes in BFP. (**C**). Changes in FMI. (**D**). Changes in Average SFT. Black and grey cross-bars indicate median values.

Physical activity attenuated the negative impact of age on body composition and body fat accumulation in middle-age women (Figure 3). Regular physical activity was linked to lower BMI, yet high body fat percentage and SFT were still observed. Snacking was associated with higher BFP (37.4% vs. 30.8%, ES = 0.37 [95% CI: 0.08–0.62], *p*_adj_ = 0.04).

### 3.4. Subcutaneous Fat Accumulation in Women with NWO

Women with NWO and overweight shared a similar SFT profile, setting them apart from those with NWNO (Table 5). In contrast to women with NWNO, women with NWO who had higher total and subcutaneous body fat, a slight weight increase was correlated with body fat accumulation rather than FFM. Similar patterns were observed in women under 45 and over 45 years, indicating consistent results across age groups. Women with NWO and overweight had similar BFP, chest, subscapular, and thigh SFT.

In contrast to women with NWNO, women with NWO had significantly elevated SFT in all body parts. The patterns remain consistent across all age groups. Significant differences in abdominal SFT (~7 mm) and minor differences in triceps (~1.7 mm) measurements were revealed between women with NWNO and NWO, as shown in Table 5.

**Figure 4:**
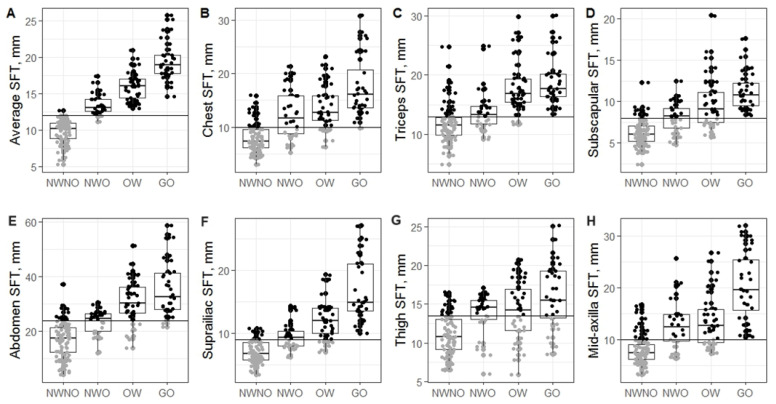
Distribution of SFT across NWNO, NWO, overweight and general obesity groups. (**A**). Average SFT. (**B**). Chest SFT. (**C**). Tricep SFT. (**D**). Subscapular SFT. (**E**). Abdomen SFT. (**F**). Suprailiac SFT. (**G**). Thigh SFT. (**H**). Mid-axilla SFT. Black circles indicate values higher than the cutoff points, grey circles—lower.

The average (mean) SFT was determined as the best predictor of NWO status in women of all ages (Table 6 and Table 7 and Figure 4). The average SFT was set at 12.0 mm based on Youden statistics. The Youden index is a maximum value serving as a criterion for determining the optimal cutoff point. Around 85% of NWO cases were determined correctly using the validation dataset (Table 6, precision rate).

SFT in chest showed lower accuracy due to higher false positive rate. Despite the biggest mean difference in abdomen SFT between women with NWO and NWNO, the accuracy and precision were lower compared to the mean SFT.

In summary, mean (average) SFT was determined as the best predictor of NWO status. Using individual SFT measurements resulted in a higher error rate in NWO status prediction.

### 3.5. Predictors of NWO

**Figure 5:**
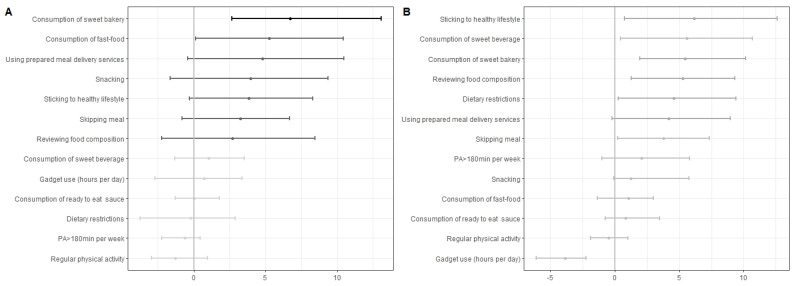
Lifestyle variable importance calculated using Boruta algorithm in the group of women under 45 years. (**A**). Classification of NWO and NWNO cases. (**B**). Classification of NWO and women with BMI ≥ 25 kg/m^2^. Black crossbars indicate predictors selected by Boruta as important (significant). Dark grey crossbars indicate tentative predictors. Light grey—unimportant (insignificant). Median minimum and maximum of mean decrease accuracy showed as error bars.

Women under 45 with NWO used meal delivery services (Figure 5A, tentative, indicated as dark grey), consumed sweet bakery items (Figure 5A, important, indicated as black, Fisher test: *p*_adj_ = 0.04), fast food (Figure 5A, tentative, indicated as dark grey), and snacks more frequently (Figure 5A, tentative, indicated as dark grey). They also skipped meals more frequently (Figure 5A, tentative, indicated as dark grey) and were less likely to stick to a healthy lifestyle (Figure 5A, tentative, indicated as dark grey) or check food composition (Figure 5A, tentative, indicated as dark grey). All predictors were associated with a small increase in BFP that was not statistically significant after implying Hochberg correction (+2%, *p*_adj_ > 0.05). In women over 45, snacking was a significant predictor for increased total body fat and linked to NWO (37.4% vs. 30.8%, effect size = 0.37 [95% CI 0.08–0.62], *p*_adj_ = 0.04). No lifestyle differences were observed among women with NWO, overweight, and general obesity (*p*_adj_ > 0.05). Women with NWO have lifestyle patterns similar to women with BMI > 25 kg/m^2^ (Figure 5B).

## 4. Discussion

This study examined body composition and lifestyle differences among women with NWNO, NWO, overweight, and general obesity, aiming to identify indicators and predictors of NWO. Women with NWO exhibit similarities in body composition, fat distribution, and frequency of consuming unhealthy food with women who were with overweight and general obesity, but not with NWNO.

The present study utilized quick and convenient ultrasound method to evaluate total and subcutaneous body fat. The 2.5 MHz amplitude (A)-mode ultrasound scanner (BodyMetrix™; BodyMetrix Pro System BX2000; Livermore, CA, USA) has undergone validation in several cohorts, confirming its effectiveness for body composition and SFT assessment [13,14,23,25,26,27]. This method demonstrates excellent test–retest and interrater reliability [11,13,14]. A-mode relies on detecting the fat-muscle interface and accurately measures SFT with a precision of 1 mm at all measurement sites. Ultrasound scanning offers advantages over caliperometry by assessing subcutaneous fat thickness without the limitations of grasping, enabling measurement in individuals with high body fat content [26]. In the present study, the JP7 formula was utilized for evaluating body composition. In women and men with normal weight (BMI < 25 kg/m^2^), the mean difference between air displacement plethysmography (ADP), bioimpedance analysis (BIA) and the JP7 (BodyMetrix™) is insignificant [25]. The agreement between the 3C model and JP7 formula is lower in individuals with overweight and obesity [11]. BodyMetrix™ tends to underestimate BFP, indicating accuracy limitations for this group. [11]. The present study is the first one to employ ultrasound (A-mode) for evaluating the prevalence of NWO. The present study described a curvilinear relationship between BMI and BFP, aligning with prior research findings [18]. BFP can be predicted accurately when BMI values exceed approximately 25 kg/m^2^. BMI explained 23% of BFP variance in the group of women with BMI ≥ 25 kg/m^2^ and 19% in women with BMI under 25 kg/m^2^. Women with BMI < 25 kg/m^2^ had greater variation in BFP than women with BMI ≥ 25 kg/m^2^. Greater discrepancies of BFP at lower BMI values may be associated with cases of NWO. Although BMI is a common measure of adiposity, relying solely on it tends to underestimate the prevalence of obesity, as it does not account for body composition variations. Individuals with normal weight but increased adiposity have a higher risk of metabolic syndrome and its components than their counterparts with normal weight without obesity [3,4,5,6,7,8,9,10]. Comprehensive examination, including body composition analysis and evaluation of body fat distribution, is recommended in all nutritional status groups. When using a 30% BFP threshold, the overall NWO prevalence was nearly 20%. One in four women with BMI < 25 kg/m^2^ had BFP exceeding 30%. Previous studies reported notable prevalence of NWO in children, adolescents, and individuals under 30 years [2,8]. At a young age, NWO is already associated with a high risk of metabolic disorders [8].

BMI shows a curvilinear relationship with age, while total body fat increases linearly [28,29]. BMI underestimated adiposity in women over 45 years due to significant changes in body composition. Body composition shifts with age, showing increased total body fat accumulation and decreased fat-free mass in middle-aged (over 45 years) women. After adjusting for nutritional status, the analysis showed no significant age-related differences in subcutaneous fat accumulation, suggesting body fat redistribution and visceral fat accumulation. Aging leads to a loss of fat-free mass, primarily muscle mass, resulting in decreased strength and mobility, which leads to an increase in body fat accumulation and potential health risks. Muscle mass peaks in the fourth decade and subsequently declines [29]. During this age period, weight gain is primarily linked to the accumulation of body fat, specifically visceral fat [29]. A longitudinal study found significant increases in body weight and total body fat after age 45, while subcutaneous fat levels showed variability, either increasing or decreasing [30]. Increases in fat mass with aging are accompanied with a redistribution of fat from extremity to trunk and visceral fat accumulation [30]. In the present study, SFT in extremes and trunk were mostly associated with nutritional status rather than with age. In women over 45, elevated TBF may be linked to the rising prevalence of NWO and general obesity. A study of 564,254 adult women in Russia revealed that general obesity prevalence nearly doubled by age of 50–64 years, indicating significant age-related increases in obesity rates [31]. Adverse age-related changes in body composition, such as increased body fat, particularly visceral fat, and decreased fat-free mass, are associated with higher risk metabolic abnormalities [7]. Women with BFP exceeding 37% have been identified as being at a heightened risk for metabolic syndrome [7]. As individuals age, physical activity levels decline, increasing the risk of general obesity and NWO [29]. Post-menopause, women experience unique nutritional needs that differ from those of the general population [29,32]. Adopting healthy eating habits and engaging in regular resistance training can mitigate age-related changes in body composition, promoting better health outcomes in women over 45 years. In the present study, women over 45 years who did at least 180 min of physical exercise per week and did not snack frequently had significantly lower TBF, subcutaneous body fat and risk of NWO.

A slight increase in body weight in women with NWO was primarily linked to body fat accumulation, but not to fat free mass. They showed no significant differences in FFMI and FFM compared to individuals with NWNO. Subcutaneous fat levels rise with TBF accumulation and typically exhibit an even distribution throughout the body. Commonly used obesity criteria like BMI, waist circumference, and waist-to-hip ratio may not capture cases of NWO. Measuring SFT in addition to body composition analysis can be useful in identifying NWO cases. Women with NWO exhibit similarities in SFT profile with women who are overweight and those with general obesity, rather than with NWNO. The mean value of SFT, calculated as the average of measurements at seven sites, was selected as the best predictor of NWO. Youdin’s statistic was used to determine threshold to effectively distinguish between NWO and NWNO cases in the studied population. The mean SFT was set at 12 mm, aligning with the 66th percentile. The precise level was calculated as 85%. Assessing regional subcutaneous fat, along with total or visceral fat, may be essential for identifying NWO and associated health conditions, especially in women. Higher trunk fat accumulation, both subcutaneous and visceral, is associated with a higher risk of type 2 diabetes in women [33]. Midthigh SFT beneath the fascia was positively associated with diabetes mellitus [34]. Individual SFT measurements can be applied; however, accuracy and precision are lower than with mean SFT.

Women with NWO shared similarities in lifestyle with women with general obesity. The frequency of consumption of sweet bakery items, mass-produced food, snacks and sweet beverages, were comparable between women with NWO and general obesity. Previous studies revealed an association of unhealthy dietary patterns with NWO risk [18]. Individuals with NWO consumed more foods high in fat and sugar, with lower consumption of fish, cereals, root vegetables, nuts, seeds, and fruits. The present study identified variations in lifestyle behaviors based on age, emphasizing the significance of considering age-related differences in lifestyle-body composition association. Unfavorable behaviors, such as meal skipping, frequent snacking, and consuming mass-produced food and sweet bakery items, were noted in women in the age period of emerging adulthood (18–29 years). During emerging adulthood, women transition from high school to university and full-time jobs, leading to significant lifestyle changes [35]. This period presents challenges related to diet, nutrition, and physical activity changes, as women have new responsibilities. Women in emerging adulthood are experiencing a decline in fruit and vegetable intake, while their consumption of confectionery, sugar-sweetened beverages, and snacks is increasing, reflecting changing dietary patterns [35]. All these factors can lead to rapid change in weight and body composition. Unhealthy behaviors in this period may result in body fat accumulation, raising the likelihood of developing obesity and related health conditions later in life. In contrast to NWNO, NWO doubles the risk of developing type 2 diabetes, underscoring the importance of addressing body composition and metabolic health beyond just weight status [3,4,5,6,7,8,9,10].

The present study has several limitations. First, the sample size of women with NWO and general obesity is low. The NWO and general obesity groups consisted of 30 and 34 individuals, respectively. Future studies should include larger sample sizes to validate the findings. The study did not analyze the association between lifestyle and NWO separately in age groups, as there was a low prevalence of NWO in younger groups of women. Instead, the analysis was conducted on combined age groups (18–23, 24–29, 30–45 together). It should be noted that no notable differences in lifestyle between these age groups were found. The second limitation pertains to the method used for evaluating BFP, which can lead to bias in NWO prevalence. In women with normal weight (BMI < 30 kg/m^2^), ultrasound in BFP estimation was more accurate than air displacement plethysmography (ADP) and bioelectrical impedance analysis (BIA) in relation to the DEXA techniques [17]. The bias in BFP estimation did not exceed 2%. However, reproducibility should be confirmed through comparison with DEXA or other established methods.

## 5. Conclusions

SFT measurements via A-mode can serve as a valuable additional tool for evaluating NWO. BMI is an unreliable indicator of adiposity in women with normal weight (BMI < 25 kg/m^2^). Almost 30% of women with a high body fat percentage (BFP ≥ 30%) were misclassified when BMI was used to measure adiposity. Women with NWO exhibit closer similarities in body composition and subcutaneous fat accumulation with those who are with overweight or general obesity, rather than with individuals with NWNO. Mean SFT accurately classified 85% of women with NWO. While age did not significantly affect subcutaneous fat accumulation, total fat levels increased with age, indicating redistribution of fat and higher visceral fat level. Engaging in regular physical activity and reducing snack consumption effectively countered age-related changes in body composition. Women under 45 years who consumed sweet bakery items, fast food, and snacks more frequently showed higher BFP and NWO status. Prevention strategies should focus on monitoring body composition and promoting healthy behaviors, particularly among young women transitioning into adulthood and women over 45 years.

## Figures and Tables

**Table 1:** Statistical procedures applied in the study.

Purpose	Statistic Procedure	Program and Package
Exploratory data analysis: assessment of data distribution and its correspondence to normal distribution.	Graphical method (normal probability plot, Q–Q plot), Shapiro–Wilk test	R environment (version 4.0.3) and Past (version 4.16)
Detecting curvilinear relationship between BMI and BFP; linear relationship between BFP and age (calculation of R^2^_adj_)	Graphical method, linear and polynomial regression models; linear and curvilinear models were compared with ANOVA function	R environment (version 4.0.3): ‘ggplot2’ package
Assessing prevalence of NWO and general obesity; effect of age on NWO prevalence; distribution of lifestyle between age groups	Fisher’s exact test and 95% confidence intervals (95% CI) calculation	Past (version 4.16) and R environment (version 4.0.3): ‘confintr’ package
Comparing body fat accumulation and body composition between age and nutritional status groups	Wilcox test, calculation of effect size and its 95% CI	R environment (version 4.0.3): ‘rstatix’ package
Multiple testing correction	Hochberg correction	R environment (version 4.0.3): basic
Prediction of NWO based on SFT measurements	ROC analysis and random forest algorithm. Area under the curve (AUC) and 95% CI were calculated. Calculation of accuracy and precision rates.	easyROC program (http://biosoft.erciyes.edu.tr/app/easyROC/; accessed on 15 June 2024)
Determining SFT cutoff points for NWO identification	Yoden’s J statistic	easyROC program (http://biosoft.erciyes.edu.tr/app/easyROC/; accessed on 15 June 2024)
Determining eating habits that can be used for differentiation of NWO from NWNO and general obesity	Boruta algorithm	R environment (version 4.0.3): ‘Boruta’ and ‘ggplot2’

**Table 2:** Distribution of nutritional status groups based on BMI and BFP values.

Statistics	18–23 Years (*n* = 28)	24–29 Years (*n* = 34)	30–44 Years (*n* = 78)	45–65 Years (*n* = 44)	Overall (*n* = 184)
Normal weight (BMI < 25 kg/m^2^; BFP from 15.6 to 37.1%)					
% [95% CI]	75 [55–88]	68 [56–85]	62 [53–74]	41 [25–54]	60 [53–73]
N	21	23	48	18	110
Normal weight without obesity (BMI < 25 kg/m^2^; BFP < 30%)					
% [95% CI]	68 [46–82]	59 [34–66]	43 [30–52]	16 [7–30]	44 [32–54]
N	19	20	34	7	80
Normal weight obesity (BMI < 25 kg/m^2^; BFP > 30%)					
% [95% CI]	7 [0–23]	9 [1–20]	18 [1–26]	25 [14–40]	16 [6–35]
N	2	3	14	11	30
Overweight (BMI ≥ 25 kg/m^2^; BFP > 30%)					
% [95% CI]	18 [8–40]	18 [14–37]	27 [15–34]	20 [10–35]	22 [11–38]
N	5	6	21	9	41
General obesity (BMI ≥ 30 kg/m^2^ and BFP > 30%)					
% [95% CI]	7 [1–23]	14 [4–37]	12 [6–21]	39 [24–55]	18 [7–35]
N	2	5	9	17	33

Notes: %—prevalence, N—sample size, 95% CI—95% confidence intervals, BMI—body mass index, BFP—body fat percentage.

**Table 3:** Body composition variations observed in women across different age groups.

Estimates	18–23 Years (*n* = 28)		24–29 Years (*n* = 34)		30–44 Years (*n* = 78)		45–65 Years (*n* = 44)	
	Me	Q1–Q3	Me	Q1–Q3	Me	Q1–Q3	Me	Q1–Q3
Weight, kg	60.3 ^▼^	56.0–70.0	58.8 ^▼^	52.3–65.8	64.5	57.0–77.5	76.4	62.0–85.8
Height, cm	167.6	164.0–171.5	163.5	160.0–168.1	166.0	162.0–172	163.4	15.9–168.7
BMI, kg/m^2^	21.1 ^▼^	19.7–25.3	21.9 ^▼^	19.7–25.9	23.1 ^▼^	21.1–26.5	28.5	22.9–31.6
FFM, kg	44.5	42.3–48.5	42.6	38.4–45.7	45.5	41.3–50.3	48.0	41.9–53.4
FFMI, kg/m^2^	16.0	14.7–17.1	15.8	14.4–16.8	16.2	14.6–17.7	17.9	15.6–20.1
FM, kg	16.5 ^▼^	14.4–23.4	16.7 ^▼^	12.6–24.1	18.9 ^▼^	15.7–27.2	28.4	20.1–33.1
FMI, kg/m^2^	5.8 ^▼^	5.1–7.6	5.8 ^▼^	4.6–7.9	6.8 ^▼^	5.6–9.3	9.9	6.7–12.3
BFP, %	26.4 ^▼^	23.4–30.2	27.8 ^▼^	23.1–33.6	28.7 ^▼^	25.1–34.8	36.3	31.8–38.7
Chest SFT, mm	9.0 ^▼^	7.3–11.2	8.9 ^▼^	6.7–14.3	9.5 ^▼^	6.6–12.8	13.6	11.1–16.6
Subscapular SFT, mm	6.2 ^▼^	5.3–7.9	6.7 ^▼^	5.1–8.3	7.5 ^▼^	6.0–9.1	9.6	7.8–12.2
Mid-axilla SFT, mm	8.6 ^▼^	7.0–12.9	7.9 ^▼^	6.9–11.4	10.0 ^▼^	7.3–14.1	14.9	10.6–21.6
Abdomen SFT, mm	21.7	12.6–25.2	20.6	12.9–32.3	22.1	16.5–30.0	25.5	21.3–29.0
Suprailiac SFT, mm	8.2	6.2–9.0	7.8 ^▼^	6.0–11.1	9.4	6.9–12.8	11.8	8.6–13.8
Triceps SFT, mm	13.0	10.5–17.1	11.8	9.9–16.3	14.0	11.7–16.4	15.7	12.9–19.3
Thigh SFT, mm	11.3	9.3–13.1	12.1	10.7–14.1	13.1	9.6–15.3	14.3	10.8–17.0
Sum of 7 SFT	77.1 ^▼^	65.6–92.6	77.6 ^▼^	59.8–103.9	87.9 ^▼^	70.5–111.1	111.6	87.4–129.7
Mean SFT (7 sites)	11.0 ^▼^	9.4–13.2	11.1 ^▼^	8.5–14.8	12.6	10.1–15.9	15.9	12.5–18.5

Notes. ^▼^—lower values compared to values of middle-age women (*p*_adj <_ 0.05 after Hochberg correction), N—sample size, BMI—body mass index, FFM—fat-free mass, FFMI—fat free mass index, FM—fat mass, FMI—fat mass index, BFP—body fat percentage, SFT—subcutaneous fat thickness.

**Table 4:** Distribution of lifestyle factors across age groups.

Lifestyle	18–23 Years (*n* = 28)	24–29 Years (*n* = 34)	30–44 Years (*n* = 78)	45–65 Years (*n* = 44)
Sticking to healthy lifestyle (Yes), % [95% CI]	48 [31–64]	43 [34–54]	58 [39–77]	43 [29–59]
Reviewing food composition (Yes), % [95% CI]	45 [26–64]	33 [19–49]	48 [37–59]	33 [19–48]
Using meal delivery services (Yes), % [95% CI]	59 ^▲^ [39–76]	63 ^▲^ [46–77]	45 ^▲^ [34–57]	7 [1–18]
Any dietary restrictions (Yes), % [95% CI]	18 [9–36]	15 [8–25]	27 [13–47]	20 [9–34]
Mean meal frequency, times	3	4	3	4
Skipping meal (Yes), % [95% CI]	21 [9–36]	8 [8–16]	8 [1–22]	13 [5–26]
Snacking (Yes), % [95% CI]	55 [38–70]	53 [42–65]	35 [18–54]	37 [23–53]
Fast-food consumption (Yes), % [95% CI]	76 ^▲^ [59–87]	78 ^▲^ [64–86]	60 ^▲^ [39–76]	20 [9–34]
Sweet beverage consumption (Yes), % [95% CI]	52 [36–67]	55 [43–66]	48 [29–68]	30 [17–46]
Consumption of sweet bakery (Yes), % [95% CI]	90 ^▲^ [76–97]	85 ^▲^ [75–91]	63 [42–79]	59 [43–73]
Consumption of sauce (Yes), % [95% CI]	72 [53–87]	73 [56–85]	58 [47–69]	50 [35–65]
Regular PA (Yes), % [95% CI]	32 [53–87]	29 [56–85]	31 [21–42]	20 [35–65]
PA ≥ 180 min per week, (Yes), % [95% CI]	21 [2–28]	15 [2–24]	20 [6–21]	11 [3–22]
Gadget use per week, hours [95% CI]	6 [5–10]	5 [3–10]	8 [5–9]	5 [3–9]

Notes. ^▲^—higher prevalence compared to middle-age women (*p*_adj_ < 0.05 after Hochberg correction), %—prevalence, [95% CI]—95% confidence intervals.

**Table 5:** Differences in body composition in women with NWNO, NWO, overweight and general obesity.

Statistics	NWO, *n* = 30	NWNO, *n* = 80	OW, *n* = 41	GO, *n* = 33	SE [95% CI]		
					vs. NWNO	vs. OW	vs. GO
Body weight, kg							
Me	62.5	58.8 ^▼^	76.5 ^▲^	88.4 ^▲^	0.37	0.71	0.84
Q1–Q3	59.8–66.8	53.8–62.2	69.8–80.4	84.38–97.5	[0.20–0.50]	[0.59–0.80]	[0.79–0.86]
Body height, cm							
Me	165.5	165.0	166.1	163.0	0.03	0.02	0.10
Q1–Q3	161.3–169.7	162.0–170.1	161.1–171.8	159.5–168.7	[0.01–0.21]	[0.01–0.28]	[0.01–0.40]
BMI, kg/m^2^							
Me	22.8	20.5 ^▼^	26.8 ^▲^	33.1 ^▲^	0.45	0.85	0.85
Q1–Q3	21.6–23.7	19.5–22.3	25.9–28.5	31.3–35.7	[0.30–0.60]	[0.80–0.86]	[0.82–0.86]
Body fat percentage, %							
Me	31.7	26.1 ^▼^	35.2 ^▲^	38.7 ^▲^	0.74	0.54	0.83
Q1–Q3	30.8–33.3	23.4–27.6	33.3–36.5	37.5–40.0	[0.64–0.81]	[0.34–0.69]	[0.77–0.86]
Fat Mass Index, kg/m^2^							
Me	7.2	5.7 ^▼^	9.5 ^▲^	12.8 ^▲^	0.70	0.79	0.86
Q1–Q3	6.7–7.7	4.9–6.1	8.7–10.2	12.0–14.2	[0.61–0.78]	[0.69–0.84]	[0.82–0.87]
Fat Free Mass Index, kg/m^2^							
Me	15.6	15.7	17.8 ^▲^	20.7 ^▲^	0.09	0.83	0.86
Q1–Q3	14.8–16.0	14.7–16.5	17.0–18.4	19.3–22.1	[0.07–0.26]	[0.76–0.85]	[0.82–0.87]
Abdomen SFT, mm							
Me	24.8	17.5 ^▼^	30.4 ^▲^	32.3 ^▲^	0.45	0.53	0.65
Q1–Q3	20.1–26.5	12.3–21.3	26.8–36.2	27.8–41.2	[0.29–0.59]	[0.35–0.68]	[0.48–0.78]
Suprailiac subcutaneous fat thickness, mm							
Me	9.3	6.8 ^▼^	12.0 ^▲^	14.9 ^▲^	0.47	0.46	0.77
Q1–Q3	8.0–10.4	5.8–8.6	10.0–14.0	13.6–20.9	[0.34–0.60]	[0.24–0.63]	[0.66–0.83]
Chest subcutaneous fat thickness, mm							
Me	11.7	7.3 ^▼^	12.7	16.0	0.47	0.16	0.45
Q1–Q3	8.8–15.8	6.1–9.5	11.2–15.8	13.6–20.1	[0.30–0.63]	[0.01–0.38]	[0.22–0.63]
Subscapular subcutaneous fat thickness, mm							
Me	8.2	6.1 ^▼^	9.1	10.7 ^▲^	065	0.26	0.67
Q1–Q3	6.8–9.2	5.2–7.0	7.5–11.1	9.6–12.1	[0.50–0.78]	[0.05–0.48]	[0.50–0.78]
Mid-axilla subcutaneous fat thickness, mm							
Me	12.5	7.5 ^▼^	12.7	19.4 ^▲^	0.46	0.06	0.52
Q1–Q3	9.8–14.9	6.2–9.2	9.5–15.8	14.2–25.2	[0.31–0.62]	[0.01–0.30]	[0.30–0.70]
Triceps subcutaneous fat thickness, mm							
Me	13.3	11.6	16.9 ^▲^	17.6 ^▲^	0.31	0.53	0.60
Q1–Q3	11.7–14.7	9.9–12.8	15.4–19.3	16.4–20.2	[0.13–0.47]	[0.33–0.69]	[0.42–0.75]
Thigh subcutaneous fat thickness, mm							
Me	14.6	10.8 ^▼^	14.2	15.7	0.44	0.02	0.28
Q1–Q3	13.0–15.5	9.2–13.0	11.6–16.9	13.1–19.3	[0.26–0.61]	[0.01–0.27]	[0.04–0.50]
The average (mean) subcutaneous fat thickness measured at seven sites, mm							
Me	13.1	10.2 ^▼^	16.1 ^▲^	18.8 ^▲^	0.74	0.64	0.82
Q1–Q3	12.5–14.2	8.8–11.0	14.4–17.0	17.8–20.3	[0.65–0.81]	[0.49–0.64]	[0.50–0.85]

Notes. ^▼^—significantly lower value compared to NWO, ^▲^—significantly higher values compared to NWO (*p*_adj_ after Hochberg correction), Me—median, Q1–Q3—25% and 75% percentiles.

**Table 6:** Optimal SFT cutoff points for prediction of BFP ≥ 30% based on Youden’s statistics.

Subcutaneous Fat Thickness, mm	Overall (N_BFP≥30%_ = 81, N_BFP<30%_ = 103)	18–29 Years (N_BFP≥30%_ = 22, N_BFP<30%_ = 40)	30–44 Years (N_BFP≥30%_ = 44, N_BFP<30%_ = 34)	45–65 Years (N_BFP≥30%_ = 37, N_BFP<30%_ = 7)	Validation (N_NWO_ = 26, N_NWNO_ = 54)	
					Accuracy	Precision
Average (mean)	12.2	12.6	12.5	11.8	94%	85%
Chest	10.5	10.5	9.9	9.6	86%	89%
Triceps	14.0	14.9	14.1	13.1	93%	80%
Subscapular	7.5	8.3	7.7	8.3	79%	68%
Abdomen	24.3	24.4	24.6	22.5	71%	63%
Suprailaic	9.3	11.3	8.6	8.7	84%	57%
Thigh	13.7	13.5	12.7	13.7	70%	56%
Mid-axilla	9.9	9.5	10.1	10.1	68%	52%

**Table 7:** The area under the curve (AUC) and 95% confidence intervals calculated to evaluate accuracy of a predictive model of BFP ≥ 30%.

Subcutaneous Fat Thickness, mm	Overall (N_BFP≥30%_ = 81, N_BFP<30%_ = 103)	18–29 Years (N_BFP≥30%_ = 22, N_BFP<30%_ = 40)	30–44 Years (N_BFP≥30%_ = 44, N_BFP<30%_ = 34)	45–65 Years (N_BFP≥30%_ = 37, N_BFP<30%_ = 7)
Average	0.99 [0.98–1.00]	0.99 [0.98–0.99]	0.99 [0.98–1.00]	0.98 [0.91–0.99]
Chest	0.85 [0.98–0.94]	0.95 [0.89–0.99]	0.84 [0.77–0.94]	0.88 [0.78–1.00]
Triceps	0.86 [0.80–0.91]	0.88 [0.79–0.97]	0.84 [0.82–0.97]	0.52 [0.52–0.95]
Subscapular	0.88 [0.83–0.93]	0.90 [0.83–0.98]	0.83 [0.76–0.94]	0.89 [0.76–0.99]
Abdomen	0.89 [0.84–0.93]	0.92 [0.85–0.99]	0.94 [0.84–0.99]	0.84 [0.75–0.97]
Suprailaic	0.91 [0.88–0.96]	0.87 [0.79–0.97]	0.93 [0.90–0.99]	0.95 [0.80–0.99]
Thigh	0.82 [0.73–0.86]	0.82 [0.67–0.82]	0.81 [0.72–0.91]	0.74 [0.60–0.90]
Mid-axilla	0.87 [0.83–0.93]	0.90 [0.80–0.99]	0.80 [0.70–0.89]	0.97 [0.93–0.99]

## Data Availability

All data generated or analyzed during this study are included in this article. Further enquiries can be directed to the corresponding author.
